# Cumulative Lifetime Violence, Gender, Social Determinants of Health and Mental Health in Canadian Men: A Latent Class Analysis

**DOI:** 10.1007/s10896-023-00502-0

**Published:** 2023-02-09

**Authors:** Kelly Scott-Storey, Sue O’Donnell, Nancy Perrin, Judith Wuest

**Affiliations:** 1 Faculty of Nursing, University of New Brunswick, Fredericton, NB Canada; 2 School of Nursing, Johns Hopkins University, Baltimore, MD USA

**Keywords:** Cumulative lifetime violence, Latent class analysis, Mental health, Men, Gender role conflict, Social determinants of health

## Abstract

**Purpose:**

Among men, violence is pervasive and associated with poor mental health, but little is known about which men are most vulnerable. Our purpose is to address this gap by exploring mental health and social determinants of health (SDOH) including gender role conflict (GRC) in heterogenous groups of men with distinct patterns of cumulative lifetime violence (CLV) as target and perpetrator.

**Methods:**

Latent class analysis was conducted using means of 64 indicators of CLV severity collected from a community sample of 685 eastern Canadian men, ages 19 to 65 years. Class differences by SDOH, and depression, anxiety, and posttraumatic stress disorder (PTSD) were explored with Chi-square and analysis of variance.

**Results:**

A 4-class solution was optimal. Class 1 had the lowest CLV severity; were more likely to be better educated, employed, and have little difficulty living on their incomes; and had better mental health than other classes. Class 2, characterized by moderate psychological violence as both target and perpetrator, had mean depression and PTSD scores at clinical levels, and more difficulty living on income than Class 1. Classes 3 and 4 were typified by high severity CLV as target but differentiated by Class 4 having the highest perpetration severity, higher GRC, and being older. In both classes, mean mental health scores were above cut-offs for clinical symptomology and higher than Classes 1 and 2.

**Conclusion:**

This is the first evidence that distinct patterns of CLV severity among men intersect with GRC and SDOH and are uniquely associated with mental health.

Mental health has been described as a silent crisis affecting men differentially according to social determinants of health (SDOH), with employment instability, financial strain, lower education, family breakdown, adverse childhood experiences and masculine gender socialization being linked to poorer mental health (Affleck et al., 2018). In general, SDOH are social and economic factors that overlap to simultaneously advantage or disadvantage the health of individuals or populations (Health Canada, 2020). Among men, violence as target and/or perpetrator is a pervasive SDOH that significantly affects men’s mental health (Haegerich & Hall, 2011). Although not all men with histories of interpersonal violence have poor mental health, our grasp of *which* men are more at risk is limited. We posit this knowledge gap is due to how violence severity has been operationalized, and the limited study of intersections among patterns of violence and SDOH that may strengthen or disadvantage mental health. Commonly, the phenomenon of *cumulative lifetime* violence (CLV) is overlooked, and violence exposure is measured narrowly as one or two types (physical, psychological, or sexual) as target or perpetrator, in one context (e.g., family, workplace) in childhood or adulthood (Scott-Storey, 2011). But violent incidents rarely occur in isolation and experiences as target and perpetrator are often interconnected across the lifespan (Hamby & Grych, 2013).

Links between violence and health are studied mainly with variable-oriented methods that assume sample homogeneity and ignore the heterogeneity of men’s experiences and outcomes. Exploration of heterogeneous patterns of violence holds promise for identifying men vulnerable for mental health disparity by economic and social characteristics including gender. Gender role socialization influences how men experience and respond to violence (Fallot & Bebout, 2012). Men internalize socially prescribed masculine roles, beliefs, and behaviors as standards for their own actions and emotions and may experience gender role conflict (GRC) when these standards constrain feelings or actions (O’Neil & Denke, 2016). GRC has been associated with poorer mental health (O’Neil, 2008). Knowledge of differences in mental health and SDOH by pattern of CLV severity (CLVS) is vital for identifying those men most in need of trauma- and violence-informed (TVI) care and the development of prevention and intervention strategies. We used data from the Men’s Violence Gender and Health Study of eastern Canadian men to begin to address these gaps.

## Background

The case for examining CLV’s association with men’s mental health is substantiated by evidence that similar etiological pathways such as biological stress response and poverty underpin both victimization and perpetration across the lifespan (Hamby & Grych, 2013). As well, the likelihood of accumulating CLV exposure is supported by empirical findings from individual studies and systematic reviews showing the range of contexts where boys and/or men who are targeted for specific types of violence endure subsequent adverse mental health. For example, physical, sexual and emotional abuse has been associated with depression and/or anxiety over the life course (Hillberg et al., 2011; Lindert et al., 2014); verbal bullying with depression in adolescents (Turner et al., 2013); past year community physical violence with mental health in young men (Olofsson et al., 2009); childhood institutional physical and sexual abuse with posttraumatic stress disorder (PTSD) and mood disorders in adulthood (Wolfe et al., 2006); dating violence with depression (Kaura & Lohan, 2007); intimate partner violence (IPV) with depression, PTSD, and anxiety (Scott-Storey et al., 2022); adult sexual assault with anxiety and depression (Peterson et al., 2011); and workplace bullying with panic attacks, depression and PTSD (O’Donnell & Macintosh, 2015). Less attention has been paid to the mental health of male perpetrators of violence in the general population, despite most violent crime being attributed to men (Haegerich & Hall, 2011). Research has focused on specific groups; for instance, violence perpetration has been linked to anxiety among gang members (Coid et al., 2013); PTSD among police officers who have injured or killed (Komarovskaya et al., 2011); and PTSD, depression, and anxiety among those with combat injuries (Stevelink et al., 2014). Overall, this evidence suggests that mental health is linked to discrete violent incidents as target or perpetrator.

But there is growing evidence demonstrating accumulating patterns of lifetime violence, particularly associations between child maltreatment and subsequent adult violence perpetration and/or victimization (Godbout et al., 2019). Associations between such patterns and mental health also have been uncovered. For example, Affiifi et al. (2009) found that men who experienced childhood sexual abuse were three times more likely to be targeted for IPV; those who later experienced IPV were twice as likely to have two or more past year psychiatric disorders than those without IPV. In another study, older male and female survivors of the Swiss child welfare system, compared to peers not in the system, reported more severe child maltreatment associated with higher rates of lifetime trauma including physical and/or sexual assault in adulthood as well as higher lifetime burden of anxiety, depression, and PTSD (Thoma et al., 2021). Despite this evidence of mutually-reinforcing, cyclical relationships between violence and mental health across the lifespan, credibility of these findings is threatened because measurement of violence is rarely comprehensive (Scott-Storey, 2011). Our 64-item CLVS scale for men is an inclusive measure of severity of physical, psychological, and sexual violence in childhood through to adulthood, as target and perpetrator, in families, schools, workplaces and communities (Scott-Storey et al., 2020). Total CLVS-64 scores were found to be significantly related to total scores for depression, PTSD, and anxiety in a sample of 590 men (Scott-Storey et al., 2018). A limitation of these findings was use of variable-oriented methods that neglected the heterogeneity of experiences of violence severity among men in the sample.

Latent class analysis (LCA) is a person-oriented method that configures heterogeneity among cases on variables of interest by identifying classes, each with unique common patterns of characteristics distinct from other classes (Nurius & Macy, 2008). An LCA of CLVS as target and perpetrator in a community sample of men may uncover heterogeneity in patterns of lifetime violence as a basis for probing differences in mental health and SDOH. Class differences in mental health by patterns of violence have been explored in other LCA studies. For example, LCA was used to identify four classes of men’s *IPV victimization* and their association with depression or a chronic mental health disability (CMHD; Carbone-López et al., 2006). Compared to the *No Violence* class (91.7%), the *Interpersonal Conflict* class (3.4%) had odds of 1.9 for depression and 2.9 for CMHD, the *Systematic Abuse* (sustained force, stalking) class (1.6%) had odds of 2.4 for depression and 2.9 for CMHD, and the *Physical Aggression* (without force, not sustained) class (3.3%) had mental health similar to the *No Violence* class. In another LCA, Burns et al. (2016) explored the patterns of CLV *victimization* and their relationship to mental health. Compared to the *Low Interpersonal Victimization* class (81.4%), the *High Lifetime Victimization* class (2.1%) had odds of 8.0 for anxiety, 7.3 for depression, and 12.6 for PTSD. The *High Witnessing Domestic Violence & Polyvictimization* (4.5%) and the *High Adult Victimization* (12%) classes each had odds ratios two to three times higher than the *Low* class for these conditions. In all classes, men with lower socio-economic status were twice as likely to have anxiety, depression, and PTSD than those who were not, and Black and Hispanic men were significantly more likely to have anxiety and depression than Caucasian men. However, no comparison of differences between classes on these SDOH were reported.

Davis et al. (2018) used LCA to identify four patterns of child maltreatment in young men and their differential perpetration of adult *IPV perpetration, peer violence* and *sexual assault* and examined class differences by both mental health and SDOH. In comparison to the *Low* maltreatment class (80%), the *High Emotional and Physical* class (12%) had the highest rates of physical and psychological IPV perpetration and the second highest mean score on depression. The *Emotional and Sexual* class (4%) had the second highest rate of psychological IPV perpetration, the highest rate of sexual assault and the second highest mean anxiety score. The *Polyvictimization* class (4%) had moderate rates of physical and psychological IPV perpetration and the highest rates of anxiety and depression as well as significantly lower education and income with more than half being men of color. These study results suggest that LCA is a promising strategy for exploring which men may be most at risk for poor mental health by patterns of CLV and SDOH. Notably, we found no studies that had used LCA analysis to pinpoint *lifetime* patterns of *both* violence victimization and perpetration among men, despite their common etiological underpinnings (Hamby & Grych, 2013). Nor did we find LCA studies that explored GRC by CLV class for identifying vulnerability to mental health problems. Our objective in the present analysis was to identify men most vulnerable for mental health problems by patterns of CLV, GRC and SDOH in a community sample of eastern Canadian men. Our goals were to: (a) identify classes of men with distinct patterns of CLV, (b) explore differences among classes by GRC and SDOH, and (c) identify differences in mental health (depression, anxiety, PTSD) among classes while controlling for GRC and SDOH.

### Methods

We analysed data collected from April 2016 until March 2018 after receiving ethical approval from the University of New Brunswick Research Ethics Board. Using community contacts and online classified advertisements, we recruited a community convenience sample of 685 individuals who self-identified as men and met inclusion criteria of being English-speaking, age 19 to 65 years, a resident of eastern Canada and willing to take part in a study of violence, gender, and health. Those recruited were sent the letter of information and an online link for eligibility and consent; following consent they were sent a link to the online survey. Upon survey completion, men were directed to a debriefing page with strategies and resources to manage any distress experienced from taking part. Each received a 20 Canadian dollar (CAD) honorarium.

## Measures

The online survey included established scales and self-report questions.

### Cumulative Lifetime Violence Severity

CLV was measured with severity scores (range 1 to 4) on each item (see Table 1 for items) of the CLVS-64 scale (Scott-Storey et al., 2020). On 4-point scales, perceptions of *how often* (1 = never, 4 = often) each experience of violence occurred and *how distressing* it was (1 = not at all, 4 = very) were scored. For each item, frequency and distress scores were summed and averaged for *severity* scores (range 1 to 4). Severity scores for the 64 items were summed and averaged for a total CLVS-64 score between 1 and 4 with higher scores reflecting greater CLV severity. Internal consistency was α =0.95.

**Table 1:** Mean Cumulative Lifetime Violence Severity  (CLVS) 64 item severity scores by class (N = 685)

CLVS-64 Items	CLASS 1: Moderate Psychological, Target—Low Perpetrator All	CLASS 2: High Psychological Target—Moderate Psychological Perpetrator, Child & IPV	CLASS 3: Highest Target All Types—Moderate Psychological Perpetration	CLASS 4: High Psychological & Physical, Moderate Sexual Target—Highest Perpetrator All Types
	n = 336	n = 235	n = 48	n = 66
*Child Target*				
I was yelled at, taunted, put down, picked on, isolated, or scared by someone with power over me (such as, parent, caregiver, teacher, coach, or someone older).	1.95	3.14	3.74	3.39
I was hit, kicked, slapped, burned, choked, or otherwise physically hurt by someone with power over me (such as, parent, caregiver, teacher, coach, or someone older)	1.41	2.39	3.31	2.73
I was touched against my will in a sexual way or forced or pressured into sexual activity by someone with power over me (such as, parent, caregiver, teacher, coach, or someone older).	1.08	1.50	2.11	1.81
I saw violence (such as bullying, threats, physical or sexual assault, or harassment) among my family members, or those I lived with.	1.44	2.33	2.99	3.00
I saw violence (such as bullying, threats, physical or sexual assault, or harassment) in my community involving others.	1.90	2.80	3.36	3.16
As a part of a team or group, I was the target of criticism or comments from another child/peer that felt uncomfortable or that ‘crossed the line.’	1.39	2.49	3.36	2.80
As part of a team or group, I was physically threatened or physically hurt by another child/peer in a way that ‘crossed the line.’	1.22	2.02	2.97	2.60
As part of a team or group, I was touched against my will in a sexual way or forced/pressured into sexual activity by another child/peer.	1.01	1.09	1.56	1.48
I was the target of messages or photos meant to hurt, scare, control, or put me down (such as written notes, texts, or social media).	1.11	1.48	2.57	1.83
I was harassed or stalked.	1.21	1.76	3.06	2.20
I was yelled at, put down, isolated, made to feel afraid or controlled by someone I dated.	1.19	1.65	2.77	2.09
I was hit, kicked, slapped, burned, choked, or otherwise physically hurt by someone I dated.	1.04	1.36	2.11	1.46
I was touched against my will in a sexual way or forced/pressured into sexual activity by someone I dated.	1.04	1.11	1.82	1.30
At school, home or in the community (other than in a dating relationship or within a team/group), I was yelled at, taunted, isolated, put down, picked on, or scared by another child/peer.	1.61	2.83	3.57	2.92
At school, home or in the community (other than in a dating relationship or within a team/group), I was hit, kicked, slapped, burned, choked, or physically hurt by another child/peer.	1.31	2.35	3.19	2.80
At school, home or in the community (other than in a dating relationship or within a team/group), I was touched against my will in a sexual way or forced or pressured into sexual activity by another child/peer.	1.03	1.18	1.88	1.61
*Adult Target*				
As a part of a team or group, I was the target of criticism or comments that felt uncomfortable or that ‘crossed the line.’	1.28	1.86	2.79	2.62
As a part of a team or group, I was physically threatened or physically hurt in a way that ‘crossed the line.’	1.10	1.48	2.49	2.39
As part of a team or group, I was touched against my will in a sexual way or forced/pressured into sexual activity.	1.01	1.05	1.55	1.36
In a dating or partner relationship, I have been yelled at, put down, isolated, controlled, or made to feel afraid.	1.48	2.29	3.01	2.55
In a dating or partner relationship, I have been hit, kicked, slapped, burned, choked or otherwise physically hurt	1.17	1.74	2.50	2.11
In a dating or partner relationship, I have been touched against my will in a sexual way or forced/pressured into sexual activity	1.03	1.15	1.95	1.38
At work I have been put down, overly criticized, controlled, isolated, or made to feel small.	1.54	2.34	3.07	2.93
At work I have been taunted, called names, or treated meanly, based on my gender, sexual orientation, or other qualities.	1.09	1.57	2.84	1.96
At work I have been physically threatened, pushed, shoved, or had things thrown at me by a boss or co-worker.	1.08	1.30	1.90	1.90
I have experienced physical violence due to the nature of my work, for example, military, policing, health care.	1.25	1.50	1.77	2.34
At work I have been harassed or touched against my will in a sexual way or forced/pressured into sexual activity.	1.01	1.06	1.35	1.16
Other than in dating or partner relationships, at work or within teams/groups, I have been yelled at, put down, isolated, controlled, or made to feel afraid.	1.20	1.91	3.00	2.20
Other than in dating or partner relationships, at work or within teams/groups, I have been physically threatened or experienced physical violence in public places, such as on the street, in bars, at sporting events or concerts.	1.28	1.84	2.74	2.49
Other than in dating or partner relationships, at work or within teams/groups, I have been touched against my will in a sexual way or forced/pressured into sexual activity.	1.04	1.11	1.67	1.39
I have been hit, kicked, slapped, burned, choked, or otherwise physically hurt by a caregiver or family member (other than a partner).	1.05	1.34	2.01	1.89
I have been the target of messages or photos that were meant to hurt, scare, control, or put me down (such as written notes, texts, or social media).	1.08	1.46	2.38	1.89
I have been harassed or stalked.	1.11	1.48	2.57	2.17
I have seen violence (such as bullying, threats, physical or sexual assault, or harassment) among my family members, or those I lived with.	1.27	1.82	2.94	2.52
I have seen violence in my community (such as workplace bullying, threats, physical or sexual assault, or harassment).	1.56	2.26	3.09	2.95
I have been physically threatened or experienced physical violence in situations of unrest, such as civil and political conflicts (strikes, protests), jail, or war.	1.17	1.39	1.74	2.58
*Child Perpetrator*				
I sent written notes, texts, or messages or photos by social media to hurt, put down, scare, or control another person.	1.15	1.27	1.60	1.86
I harassed or stalked another person.	1.07	1.20	1.41	1.83
As a part of a team or group, I criticized, or made comments that made someone feel uncomfortable or that ‘crossed the line’.	1.24	1.64	1.88	2.20
As a part of a team or group, I physically threatened or physically hurt someone in a way that ‘crossed the line’.	1.13	1.34	1.32	2.55
As a part of a team or group, I touched someone against their will in a sexual way or forced/pressured someone into sexual activity by using threats, physical force, pressure, or drugs/alcohol.	1.01	1.01	1.00	1.37
I yelled at, put down, isolated, controlled or threatened someone I dated.	1.11	1.53	1.64	2.25
I hit, kicked, slapped, burned, choked, or otherwise physically hurt someone I dated.	1.02	1.12	1.38	1.63
I made someone I dated take part in sexual activity or have sex against their will by using threats, physical force, pressure, or drugs/alcohol.	1.01	1.04	1.01	1.30
At school, home or in the community (other than in a dating relationship or within a team/group), I yelled at, taunted, isolated, left out, put down, picked on, controlled, or threatened someone.	1.24	1.70	1.73	2.47
At school, home or in the community (other than in a dating relationship or within a team/group), I physically threatened or was physically violent toward someone.	1.22	1.60	1.55	2.79
At school, home or in the community (other than in a dating relationship or within a team/group), I made someone take part in sexual activity or have sex against their will by using threats, physical force, pressure, or drugs/alcohol.	1.00	1.06	1.00	1.23
*Adult Perpetrator*				
As part of a team or group, I have criticized, or made comments that made someone feel uncomfortable or that ‘crossed the line’.	1.30	1.60	1.93	2.70
As part of a team or group, I have physically threatened or physically hurt someone in a way that ‘crossed the line’.	1.06	1.22	1.16	2.56
As a part of a team or group, I have touched someone against their will in a sexual way or forced/pressured someone into sexual activity by using threats, physical force, pressure, or drugs/alcohol.	1.00	1.01	1.00	1.27
In a dating or partner relationship, I have yelled at, put down, isolated, controlled or threatened my partner.	1.26	1.81	1.79	2.79
In a dating or partner relationship, I have hit, kicked, slapped, burned, choked, or otherwise physically hurt my partner.	1.05	1.27	1.22	1.73
In a dating or partner relationship, I touched someone against their will in a sexual way or forced/pressured someone into sexual activity by using threats, physical force, pressure, or drugs/alcohol.	1.02	1.08	1.00	1.42
At work I have put down, overly criticized, controlled, isolated, or made someone feel small.	1.13	1.34	1.52	2.37
At work I have taunted, called names, or treated someone meanly based on their gender, sexual orientation, or other qualities.	1.03	1.10	1.21	1.78
At work I have physically threatened, pushed, shoved, or thrown things at someone.	1.06	1.19	1.09	2.04
At work I have harassed or touched someone against their will in a sexual way or forced/pressured someone into sexual activity by using threats, physical force, pressure, or drugs/alcohol.	1.00	1.01	1.00	1.09
My job (for example, military, police, health care) has required me to use physical violence to control a situation.	1.24	1.35	1.45	2.20
I have sent written notes, texts or messages or photos by social media to hurt, put down, scare, or control someone.	1.08	1.26	1.42	1.86
I have harassed or stalked another person.	1.05	1.15	1.13	1.61
Other than in a dating or partner relationship, at work or as part of a team/group, I have yelled at, taunted, isolated, put down, picked on, controlled, or threatened someone.	1.10	1.37	1.56	2.30
Other than in a dating or partner relationship, at work or as part of a team/group, I have physically threatened or physically hurt someone at home, in public places, such as on the street, in bars, at sporting events or concerts.	1.09	1.37	1.40	2.40
Other than in a dating or partner relationship, at work or as part of a team/group, I have touched someone against their will in a sexual way or forced/pressured someone into sexual activity by using threats, physical force, pressure, or drugs/alcohol.	1.02	1.01	1.03	1.22
I have physically threatened or been physically violent toward someone in situations of unrest, such as civil and political conflicts (strikes, protests), jail or war.	1.10	1.17	1.14	2.35

### Social Determinants of Health

Demographic self-report items for age, education, employment status, primary occupation, pension status, sexual orientation, and geographic location were used to measure these SDOH. Difficulty living on current income was reported on a scale from 1 (none) to 5 (extremely difficult or impossible). We assessed GRC with the 37-item Gender Role Conflict Scale which measures men’s reactions to inconsistent and unrealistic gender role expectations using four 6-point subscales: success, power, and competition (GRC-SPC), restrictive emotionality (GRC-RE), restrictive affectionate behavior between men (GRC-RABBM), and conflict between work and family relations (GRC-CBWFR) (0’Neil, 2008). For each subscale, items were rated from 1 (strongly disagree) to 6 (strongly agree) and summed and averaged for a total subscale score of 1 to 6 with higher scores indicating greater GRC. Coefficient alphas for subscales have ranged from the low 70s to the low 90s (O’Neil, 2008). In this analysis, subscale internal consistencies were: GRC-SPC (α = 0.88); GRC-RE (α = 0.90); GRC-RABBM (α = 0.90); GRC-CBWFR (α = 0.85). 

### Mental Health

Depressive symptom frequency in the past 2 weeks was measured with the Center for Epidemiologic Studies Depression Revised (CESD-R) 20 item, 4-point scale (rarely to most of the time; Eaton et al., 2004). Summative scores ranged from 0 to 60, with scores greater than 15 indicative of clinical depression symptomology. The CESD-R has reliability and validity in community samples diverse by age and gender (Van Dam & Earleywine, 2011) and in this analysis, α was 0.95. We used the Generalized Anxiety Disorder 7-item 4-point scale (not at all to nearly every day) to assess anxiety symptom frequency over the previous 2 weeks. Summative scores range from 0 to 21, with scores greater than 9 being an indicator of possible clinical anxiety (Spitzer et al., 2006). Reliability and construct validity are well-established in the general population (Löwe et al., 2008) and, in this analysis, α was 0.94. Τhe degree to which men were bothered by PTSD symptoms in the past month was measured with the PTSD Checklist, Civilian Version, a 17-item, 5-point scale (not at all to extremely) and summed for scores ranging from 17 to 85 (Blanchard et al., 1996; VA National Center for PTSD, 2012). Scores of 35 or higher indicated possible PTSD. Internal consistency, test-retest reliability and convergent and discriminant validity have been established in non-clinical samples (Conybeare et al., 2012). In the current sample, α was 0.95.

### Analysis

Latent class analysis was used to identify distinct groups of men using the severity scores of 64 items on the CLVS-64 scale. LCA provides a probability of class membership for each class for each person. We examined solutions with 2–8 classes and selected the final solution based on the Akaike Information Criterion (AIC), Bayesian Information Criterion (BIC), and interpretability of the solutions. Class solutions were inspected for interpretability by conceptual dimensions of CLVS including life stage (child, adult), role (target, perpetrator), type (physical, psychological, sexual) and context (family, peer, partner, workplace, community; Scott-Storey et al., 2020). Men were classified into the class for which they had the highest probability. Chi-square or one-way analysis of variance (ANOVA) were used to test differences between the classes on social determinants of health including GRC. Bonferroni post-hoc testing was used to determine significant differences in unadjusted mean scores between classes. When the assumption of homogeneity of variance was not met for ANOVA, Welch’s F was reported and Games-Howell post-hoc testing was used. Differences between the classes on depression, PTSD, and anxiety were tested with ANOVA using both unadjusted models and models adjusted for GRC and social determinants that differed significantly by class. Analyses were conducted in STATA 16 and SPSS 26.

## Results

### Description of the Sample

Of the 685 men, 567 (82.8%) reported experiences as *both* targets and perpetrators of violence in their lifetimes, 100 (14.6%) as targets only, 3 (0.4%) as perpetrators only, and 15 (2.2%) as neither. The mean CLVS severity score was 1.44 (SD = 0.35). Men ranged in age from 19 to 65 years (M = 37.57, SD = 12.45); 612 (89.3%) identified as heterosexual; 399 (58.2%) were married or living with a partner; and 242 (35.3%) had dependent children under 18 years of age. Most men were employed (n = 472, 68.9%) and had some post-secondary education or were college graduates (n = 515, 75.3%). In the prior year, 291(42.9%) had earned less than 25,000 CAD, 161 (23.7%) 25,000 to 49,999, and the remainder 50,000 or more; 53 (7.7%) had a retirement pension. Of the sample, 105 (15.3%) had for most of their working life been employed in the military, law enforcement, security, health care or as a first responder. The majority (n = 565; 82.5%) identified as Anglophone, 52 (7.6%) as Francophone, 16 (2.3%) as Indigenous, and 51 (7.4%) as not identifying with any of these groups.

### Latent Class Analysis

**Fig. 1:**
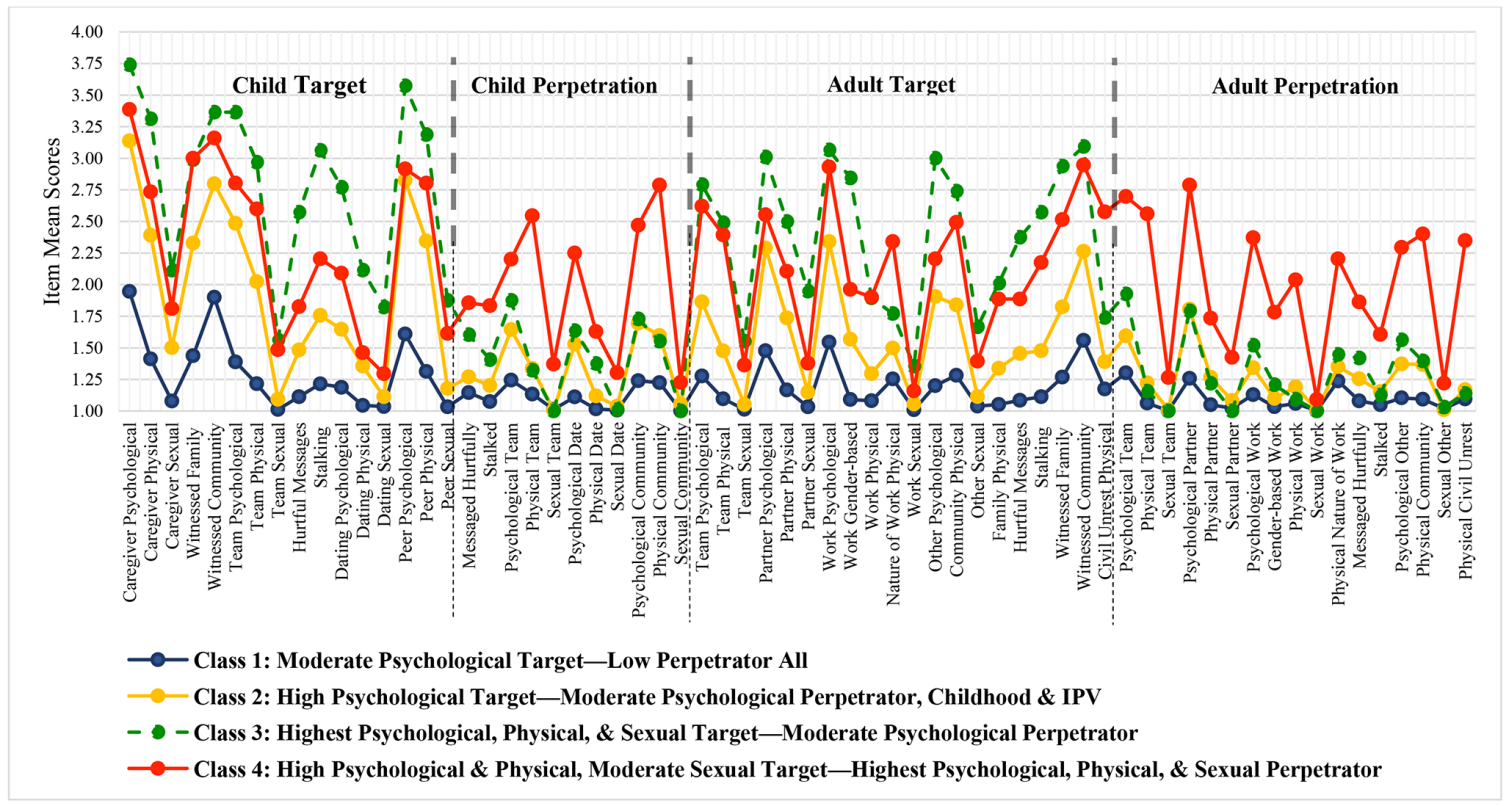
Cumulative Lifetime Violence Target & Perpetrator Severity Item Mean Scores by 4 Latent Classes (N = 685)

Solutions were estimated starting with 2 classes and the AIC and BIC were examined. Lower BIC and AIC indicate better fit of the model. As the number of classes increased from 2 to 4 classes, model fit improved with little gain after 4-classes; therefore, the 4-class solution was selected (AIC values were 80,076, 78,000, 76,684, 74,425, 73,941, 73,576, 73,153; BIC values were 80,950, 79,169, 78,148, 76,192, 75,993, 75,923, 75,793 for 2–8 classes, respectively). In examining the interpretability of different solutions, the 4-class solution was found to be illuminated by the conceptual dimensions of CLVS. Table 1 shows the mean CLVS item scores by class; Fig. 1 is a visual depiction of CLVS mean item scores by class.

### Latent Classes of Cumulative Lifetime Violence Severity

#### Class 1

This class was the largest (n = 336, 49.1%) with the lowest CLVS-64 total scores (M = 1.18, SD = 0.11) and named *Moderate Psychological Target—Low Perpetrator All*. As target, Class 1 had faced moderate severity psychological violence as children (from caregivers, and peers in homes, schools, and communities, and witnessing community violence) and adults (from partners, in the workplace, and witnessing in the community). Perpetration was very low.

#### Class 2

Labeled *High Psychological Target—Moderate Psychological Perpetrator, Childhood and IPV*, Class 2 (n = 235, 34.3%) had a mean CLVS-64 total score of 1.53 (SD = 0.13). Men had high severity psychological violence as target: (a) in childhood from adult caregivers, peers on teams/groups or in school, home, and community, and witnessing at home and in community and (b) in adulthood from partners, in the workplace, in teams/groups, and witnessing in the community. Perpetration was largely moderate psychological violence as children on teams, dates, and in the community and as adults on teams and toward partners.

#### Class 3

This class, the smallest (n = 48, 7.0%), was called *Highest Target All Types—Moderate Psychological Perpetration* and had a mean CLVS-64 total score of 1.96 (SD = 0.18). Men had the highest scores for all physical, psychological, and sexual target items except for two linked to the nature of work (e.g., military, policing, health care or as first responders) and civil unrest. They had perpetrated moderate psychological violence, most notably: (a) in childhood towards peers in teams/groups, dating partners, and others at home, school or in the community, and (b) in adulthood in partner relationships and the workplace.

#### Class 4

Named *High Psychological & Physical, Moderate Sexual Target—Highest Perpetrator All Types*, Class 4 (n = 66, 9.6%), had a mean CLVS-64 total score of 2.10 (SD = 0.30). Men had the second highest scores on all physical, psychological, and sexual target items except for violence associated with the nature of work and civil and political unrest which were the highest. Notably Class 4 had the highest violence *perpetration* (all types and contexts) across the lifespan. See Fig. 1 for depiction of differences in the dominant pattern of CLVS by class.

### Class Differences by SDOH, GRC, and Mental Health

#### Class 1

Compared to other classes, men in Class 1, *Moderate Psychological Target—Low Perpetrator All* were more likely to be better educated, employed, and have little difficulty living on their incomes (see Table 2). They reported significantly less GRC about expectations of work and family (GRC-CBWFR) than Classes 3 and 4. They also had less GRC about expressing affection toward or having physical contact with men (GRC-RABBM) and finding ways to express feelings or emotions to others in general (GRC-RE) than those in Class 4. Their mean scores for mental health problems differed significantly from other classes and were well below the cut-off scores for clinical significance at 9.59 for depression, 26.69 for PTSD and 3.41 for anxiety (see Table 3). Class 1 had the lowest proportion with probable mental health conditions (n = 86; 25.6%) and with comorbid GAD, PTSD, and depression (n = 21, 6.3%) (see Table 4).

**Table 2:** Differences Between the Classes on Social Determinants of Health including Gender (N = 685)

	TOTAL SAMPLE	CLASS 1: Moderate Psychological, Target—Low Perpetrator All	CLASS 2: High Psychological Target—Moderate Psychological Perpetrator, Child & IPV	CLASS 3: Highest Target All Types—Moderate Psychological Perpetration	CLASS 4: High Psychological & Physical, Moderate Sexual Target—Highest Perpetrator All Types	Statistical Test of Difference (df)	*p*-value
	N = 685	n = 336	n = 235	n = 48	n = 66		
Age in Years:^1^ µ (SD)	37.57 (12.46)	37.24 (12.40)	37.34 (12.16)	35.04 (12.56)	41.92 (13.01)	*F* (3, 681) = 3.49	.015
Education: n (%)							
Less than High School	53 (7.7)	13 (3.9)	27 (11.5)	4 (8.3)	9 (13.9)	χ^2^ (9) = 28.35	.001
High School Graduate	116 (17.0)	53 (15.8)	36 (15.3)	14 (29.2)	13 (19.7)		
Some Post-Secondary	195 (28.5)	94 (28.1)	63 (26.8)	14 (29.2)	24 (36.4)		
College Graduate	320 (46.8)	175 (52.2)	109 (46.4)	16 (33.3)	20 (30.3)		
Currently Employed: n (%)	472 (68.9)	258 (76.8)	156 (66.4)	22 (45.8)	36 (54.5)	χ^2^ (3) = 28.71	< .001
Difficulty Living on Income: ^2^ µ (SD)	2.49 (1.34)	2.14 (1.20)	2.64 (1.36)	3.58 (1.33)	2.89 (1.34)	*F* (3, 145.46) = 22.63*	< .001
Identifies as Heterosexual Only: n (%)	612 (89.7)	312 (93.4)	213 (90.6)	28 (58.3)	59 (90.8)	χ^2^ (3) = 56.58	< .001
Lives in Rural area or Small Town: n (%)	240 (35.1)	108 (32.2)	91 (38.7)	18 (37.5)	23 (34.8)	χ^2^ (3) = 2.68	.443
Receives Retirement Pension: n (%)	53 (7.7)	18 (5.4)	20 (8.5)	5 (3.7)	10 (18.9)	χ^2^ (3) = 8.43	.038
Primary Occupation Military, Law Enforcement, Health Care, First Response: n %	107 (15.6)	47 (14.0)	33 (14.0)	8 (16.7)	19 (28.8)	χ^2^ (3) = 9.85	.020
Gender Role Conflict: m (SD)							
Conflicts between Work and Family Relations ^3^	3.35 (1.20)	3.20 (1.14)	3.39 (1.23)	3.71 (1.35)	3.73 (1.15)	*F* (3, 679) = 23.65	.001
Restrictive Affectionate Behavior Between Men ^4^	3.06 (1.26)	2.92 (1.20)	3.07 (1.27)	3.29 (1.42)	3.57 (1.29)	*F* (3, 677) = 26.95	.001
Restrictive Emotionality ^5^	3.28 (1.17)	3.12 (1.06)	3.34 (1.15)	3.57 (1.41)	3.71 (1.37)	*F* (3, 141.5) = 5.41*	< .001
Success, Power, & Competition	3.39 (0.98)	3.34 (0.97)	3.38 (0.98)	3.40 (1.12)	3.64 (0.91)	*F* (3, 678) = 5.16	.146

^*^ Welch Statistic

^1^ Class 4 is significantly older than each of the other classes

^2^ Classes 1 and 3 are significantly different than all other classes, classes 2 and 4 are not significantly different

^3^ Class 1 is significantly different than classes 3 and 4. All other pairs are not significantly different

^4^ Class 4 is significantly different from classes 1 and 2. All other pairs are not significantly different

^5^ Class 1 and 4 are significantly different. All other pairs are not significantly different

**Table 3:** Differences between Classes on Mental Health Variables (N = 685)

	TOTAL SAMPLE	CLASS 1: Moderate Psychological, Target—Low Perpetrator All	CLASS 2: High Psychological Target—Moderate Psychological Perpetrator, Child & IPV	CLASS 3: Highest Target All Types—Moderate Psychological Perpetration	CLASS 4: High Psychological & Physical, Moderate Sexual Target—Highest Perpetrator All Types	Statistical Test of Difference	*p*-value
	N = 685	n = 336	n = 235	n = 48	n = 66		
GAD-7 ^1 2 5^	5.79 (5.81)	3.41 (4.31)	6.79 (5.82)	11.85 (5.34)	9.95 (6.28)	*F* (3, 662) = 33.26	< .001
CESD-R ^1 3 5^	15.33 (14.41)	9.59 (10.55)	17.50 (13.75)	30.59 (16.97)	25.72 (15.97)	*F* (3, 663) = 31.02	< .001
PTSD ^1 4 5^	34.07 (15.250	26.69 (9.87)	36.51 (13.94)	53.25 (15.33)	49.17 (16.56)	*F* (3, 662) = 68.47	< .001

^1^ Classes 1 and 2 are significantly different from all other classes, Classes 3 and 4 are not significantly different as indicated by Bonferroni post-hoc tests

^2^ Generalized Anxiety Disorder-7, unadjusted µ (SD).

^3^ Center for Epidemiologic Studies Depression Revised, unadjusted µ (SD).

^4^ Posttraumatic Stress Disorder Checklist, Civilian, unadjusted µ (SD).

^5^ Pattern of results is the same when analysis adjusted for social determinants of health including gender

**Table 4:** Prevalence and Pattern of Mental Health Problems by Class (N = 685)

	TOTAL SAMPLE n (%)	CLASS 1: Moderate Psychological, Target—Low Perpetrator All	CLASS 2: High Psychological Target—Moderate Psychological Perpetrator, Child & IPV	CLASS 3: Highest Target All Types—Moderate Psychological Perpetration	CLASS 4: High Psychological & Physical, Moderate Sexual Target—Highest Perpetrator All Types
	N = 685	n = 336	n = 235	n = 48	n = 66
GAD Total Cases ^1^	157 (22.9)	28 (8.3)	68 (28.9)	30 (62.5)	31 (47.0)
Depression Total Cases ^2^	269 (39.3)	72 (21.4)	112 (47.7)	39 (81.3)	46 (69.7)
PTSD Total Cases ^3^	263 (38.4)	58 (17.3)	111 (47.4)	43 (89.6)	51 (77.3)
GAD only	6 (0.9)	3 (0.9)	3 (1.3)	0	0
Depression only	46 (6.7)	25 (7.4)	19 (8.1)	0	2 (3.0)
PTSD only	31 (4.5)	7 (2.1)	16 (6.8)	3 (6.3)	5 (7.6)
GAD & Depression	3 (0.4)	0	2 (0.9)	0	1 (1.5)
Depression & PTSD	84 (12.3)	26 (7.7)	32 (13.6)	10 (20.8)	16 (24.2)
GAD & PTSD	12 (1.8)	4 (1.2)	4 (1.7)	1 (2.1)	3 (4.5)
GAD, Depression & PTSD	136 (19.9)	21 (6.3)	59 (25.1)	29 (60.4)	27 (40.9)

^1^ Generalized Anxiety Disorder indicated by a GAD-7 score > 9

^2^ Moderate or Severe Depressive Disorder indicated by a Center for Epidemiologic Studies Depression Revised score > 15

^3^ Posttraumatic Stress Disorder (PTSD) indicated by the PTSD Checklist, Civilian Version score > 34

#### Class 2

Men in Class 2, *High Psychological Target—Moderate Psychological Perpetrator, Childhood and IPV* were more likely than other classes to have less than a high school education. They were more likely to have difficulty living on income than Class 1, but less likely than Class 3. Class 2 men had significantly lower GRC-RABBM than Class 4, implying fewer concerns about expressing affection towards or touching other men. Their mean scores differed significantly from other classes on depression (17.50), PTSD (35.51) and anxiety (6.79). In Class 2, 135 (57.4%) had clinically significant mental health problems with 38 (16.2%) having only one and 59 (25.1%) having comorbid depression, anxiety, and PTSD.

#### Class 3

This class, *Highest Target All Types—Moderate Psychological Perpetration* was significantly younger (mean age = 35.04 years) than Class 4. Among classes, they were more likely to have a high school diploma as their highest level of education and to not identify as heterosexual only, and least likely to be employed; they had the greatest difficulty living on their incomes. Their mean scores on GRC-CBWFR were significantly higher than those in Class 1 but not Class 4, suggesting similar difficulty balancing demands of work and family. GRC-RABBM scores were not significantly different from those of other classes. Class 3 had the highest mean scores on depression (30.59), PTSD (53.25), and anxiety (11.85), significantly different from Classes 1 and 2 but not from Class 4. Of Class 3, 43 (89.6%) had probable mental health problems, all had depression, and 29 (60.4%) had comorbid GAD, depression, and PTSD.

#### Class 4

Men in this class, *High Psychological & Physical, Moderate Sexual Target—Highest Perpetrator All Types* were significantly older (mean age = 41.92 years) and less likely to have a university/college degree than those in other classes. They were more likely to be unemployed and to have difficulty living on their income than those in Class 1, *Moderate Psychological Target—Low Perpetrator All*, but less likely to have difficulty living on their income than those in Class 3. Compared to other classes, significantly more received a retirement pension, and worked predominantly in the military, law enforcement or health care. Class 4 men had significantly higher GRC-CBWFR and GRC-RE, that is in expressing feelings and emotions to others, than men in Class 1. Moreover, in comparison to those in Classes 1 and 2, men in Class 4 were more uncomfortable having physical contact with and expressing emotion toward men (GRC-RABBM). Class 4 men had the second highest mean scores for depression (25.72), PTSD (49.17) and anxiety (9.95), all significantly different from Classes 1 and 2, but not Class 3 (see Table 3). Class 4 had the second highest proportion of men (n = 54, 81.8%) having one or more clinically significant mental health problems). Notably, 27 (40.9%) had co-morbid GAD, depression, and PTSD, the second highest rate among classes, and 16 (24.2%) had comorbid depression and GAD.

## Discussion

These findings support the pervasive nature of violence in men’s lives (Haegerich & Hall, 2011). Importantly they also show explicit diversity of trajectories of cumulative lifetime violence within populations, beneficial for differentiating which men may be most vulnerable for violence-related health and social inequities. Previous research demonstrated that, among men, poorer mental health is associated with more severe CLV (Scott-Storey et al., 2018). Our current LCA extends this knowledge by revealing that associations with mental health differ not only by CLV severity but also by unique interconnections among multiple, accumulating, and co-occurring experiences of violence across the lifespan as target and perpetrator. No previous LCA has explored distinct patterns of CLV in a community sample of men of this age span using such a comprehensive range of violence perpetration and victimization as represented in the CLVS-64 scale. Class 1 was characterized by the lowest violence severity, largely psychological violence as target in both childhood and adulthood, and very low lifetime violence perpetration. Class 2, also distinguished by only lifetime psychological violence, exhibited more severe violence in multiple contexts as target and moderate psychological violence as perpetrator. Classes 3 and 4 were typified by lifetime higher severity of physical, psychological, and sexual violence as targets and differentiated by: (a) Class 4 perpetration of the most severe violence, all types, all contexts, and (b) Class 3 being targeted for the most severe lifetime physical, psychological, and *sexual* violence, except for physical violence connected with the nature of work and civil unrest.

Our discovery that despite distinctly different patterns of high severity CLV, Classes 3 and 4 had similar mean mental health scores and number of clinically significant mental health problems suggests that more than one pattern of lifetime violence can be associated with *substantive* mental health pathology. Our finding that Class 3, with the most severe pattern of all types of lifetime victimization and moderate psychological perpetration, had the highest clinically significant mean mental health scores extends previous LCA results that showed polyvictimization was associated with significantly greater odds of anxiety, depression, and PTSD (Burns et al., 2016). Our finding that compared to other classes, proportionally more Class 3 men did not identify as heterosexual builds on existing knowledge that, compared to heterosexual youth, sexual minority youth are more likely to experience maltreatment and bullying in families and communities, and more anxiety and depression (Chan et al., 2022). New is the implication that a wider range of severe victimization experiences across the lifespan and moderate psychological perpetration may be implicated in poorer mental health for this group of men.

Other socio-economic indicators intersected to reveal differences between classes 3 and 4. Although men in both classes were more likely to be unemployed, Class 4 had less difficulty living on their incomes than Class 3, possibly because a greater number than expected were collecting a retirement pension. Class 4 men were the oldest, significantly different in age from Class 3 men who were the youngest. Overall, intersections among SDOH, such as age, employment, and difficulty living on income and patterns of violence may be useful for identifying characteristics of men who are more vulnerable than others. Descriptive intersectional analysis for capturing simultaneous influence and overlap of multiple SDOH has been recommended as a means of understanding joint health disparity (Bowleg, 2012).

Importantly, our results show the threat to mental health among men whose CLV histories consist solely of psychological violence, extending recent LCA findings that men whose pattern of IPV victimization was characterized by extremely high emotional abuse and high physical abuse had higher scores on PTSD, depression, and anxiety than men in other classes (Lagdon et al., 2022). In our analysis, more than half of men in Class 2 had clinically significant mental health problems with a quarter having possible comorbid depression, anxiety, and PTSD. Their pattern of CLV included recurring experiences of psychological violence as children and adults, in multiple contexts as both target and perpetrator, and mean severity scores on some psychological target items were very similar to those of Class 4, *High Psychological & Physical, Moderate Sexual Target—Highest Perpetrator All Types* (see Table 1). Particularly analogous were scores on two items capturing childhood experiences of being yelled at, taunted, put down, picked on, isolated, or scared by (a) someone with power over me (e.g., parents, caregivers, teachers, coaches) or (b) peers at home, at school, or in the community. Our findings infer that even in the absence of physical and sexual violence history, mental health may be jeopardized by psychological violence as target and perpetrator that begins early in life and recurs across the lifetime.

Our findings may be the first to show differences in GRC by distinct classes of CLV. The clearest distinction is between Classes 1 (*Moderate Psychological Target—Low Perpetrator All*) and 4 where Class 4 men had significantly higher scores on GRC associated with (a) demands of family and work, (b) expression of emotion to others in general, and (c) avoidance and fear of physical contact and expressing emotion towards men. Class 4 had the highest perpetration scores for all types of violence and, in addition to the second highest target scores in general, the highest target scores on physical violence associated with the nature of their work and situations of civil unrest. In part this may be attributed to a higher percentage of Class 4 men working in military, law enforcement, first response, and health care than in other classes because GRC across domains may be fostered when men feel they deviate from the norms of masculine ideology in such work contexts. Further, our findings that Class 4 had the highest mean GRC-RE, second highest PTSD scores, as well as the second highest target scores for childhood maltreatment and most severe IPV perpetration scores are consistent with those of Gilbar et al. (2021). They found that childhood physical neglect was indirectly related to psychological IPV perpetration through PTSD and GRC-RE in a sample of men receiving treatment for IPV prevention. The intersection of GRC with the pattern of high lifetime violence perpetration and victimization may influence severity of mental health pathology, given that men in Class 4 had the second highest mean mental health scores and high comorbidity.

### Limitations

Our findings are based on a community convenience sample of 685 men, ages 19 to 65, living in eastern Canada and may not apply to samples from other populations of men. Eastern Canada has a predominantly white population, living in rural areas, small towns, and medium-sized cities. Thus, the sample lacks the ethnic diversity present in large metropolitan cities that will be addressed in future pan-Canadian studies. Another limitation is the lack of inclusion of men over 65 years of age. Our analysis was also limited by the number of SDOH measured. Going forward, measurement of more SDOH including robust indicators such as housing and food insecurity will permit a more fulsome intersectional analysis to distinguish potential for health disparity among men with CLV histories. Our cross-sectional design reflects men’s current perceptions of CLVS; a future longitudinal study will permit exploration of change in perceptions of violence severity and the degree of stability in classes of violence.

### Implications

Despite the above limitations, these findings may alert clinicians to consider intersections among social factors and patterns of CLVS that may disadvantage men’s mental health. For example, distress or discomfort with gender role expectations, midlife, and lifetime work that commonly involves use of power over others and frequently being targeted for violence may together increase risk for clinically severe mental health issues; identification as a sexual minority man, early adulthood, and *lifetime* high physical, psychological, and sexual victimization with moderate psychological perpetration may be associated with clinically significant depression, anxiety and/or PTSD. Also worthy of attention is the finding that some men at risk for mental health problems may not be readily discernable as was the case with Class 2 whose SDOH scores were unremarkable and pattern of CLVS was principally confined to lifetime moderate psychological violence. This finding reinforces the importance of clinician use of trauma- and violence-informed approaches with men and boys to facilitate prevention, early identification, and treatment of mental health. Moreover, the prevalence of CLVS as target and perpetrator as well as clinically significant mental health issues for some men in each class from this sample supports the critical need for health policy supporting development of TVI interventions for all men presenting with depression, anxiety, and/or PTSD.

## Conclusion

To our knowledge, this is the first study to use latent class analysis to identify distinct classes or groups characterized by patterns of diverse experiences of CLVS. It is also the first to examine differences in mental health, and social determinants of health including GRC by class of CLVS. Our findings, then, are unique, and although limited to the present sample, provide direction for future research to broaden applicability. The discovery that two distinct patterns of high severity CLV, distinguished largely by differences in perpetration severity, are associated with more severe mental health pathology is a new contribution that moves beyond the adage of ‘more is worse’ and compels future study. Also intriguing for subsequent inquiry is the finding that a pattern of lifetime psychological violence only, as target and perpetrator, is associated with mental health problems. Overall, our findings regarding differences in classes by GRC and SDOH provide a stepping-stone for future complex intersectional analysis, wherein the nexus of GRC, SDOH, and patterns of violence are explored to better understand which men are more vulnerable to individual and comorbid mental health problems. Findings from such studies will provide a robust foundation for health and social policy to improve the well-being of men with CLV whose mental health is most vulnerable.

## Data Availability

The data set used in this analysis is available on reasonable request from Dr. Kelly Scott-Storey kscottst@unb.ca.
